# A suspension of inactivated bacteria used for vaccination against recurrent urinary tract infections increases the phagocytic activity of murine macrophages

**DOI:** 10.3389/fimmu.2023.1180785

**Published:** 2023-08-16

**Authors:** Anja Eggers, Melissa Ballüer, Belal A. Mohamed, Roland Nau, Jana Seele

**Affiliations:** ^1^ Department of Geriatrics, Evangelisches Krankenhaus Göttingen-Weende, Göttingen, Germany; ^2^ Department of Neuropathology, University Medical Center Göttingen, Göttingen, Germany; ^3^ Department of Cardiology and Pneumology, University Medical Center Göttingen, Göttingen, Germany; ^4^ DZHK (German Centre for Cardiovascular Research), Göttingen, Germany

**Keywords:** innate immune response, vaccine, phagocytosis, cytokine induction, urinary tract infections

## Abstract

**Background:**

Urinary tract infections are a major cause of the consumption of antibiotics in humans.

**Methods:**

We studied the effect of a vaccine (StroVac®, containing inactivated bacteria and used to prevent recurrent urinary tract infections) licensed in Germany on the release of pro-inflammatory cytokines and the phagocytosis of *Escherichia* (*E.*) *coli* in primary murine macrophages and the macrophage cell line J774A.1.

**Results:**

StroVac® increased the release of the cytokines TNF-α, IL-6, IL-12/23 p40, and IL-1β and stimulated the phagocytosis of *E. coli* in a dose-dependent manner. This effect was independent of LPS as shown by the use of macrophages isolated from LPS-resistant C3H/HeJ mice. At concentrations up to 30 mg/l it was not toxic to bacteria or eukaryotic cells.

**Conclusion:**

StroVac® does not only act via the adaptive but also by stimulating the innate immune system. This stimulation may help to build trained innate immunity against bacterial pathogens involved in recurrent urinary tract infections.

## Introduction

Urinary tract infections are a major cause of the consumption of antibiotics in humans. They are caused by *Escherichia (E.) coli* in approximately 85% of cases, whereas *Klebsiella* spp., *Proteus* spp., and *Enterococcus* spp. are the causative pathogens in 5–15% (1). Recurrent urinary tract infections and successive antibiotic courses are important drivers for the development of resistance to antibiotics (2, 3). For this reason, some guidelines prefer an immunoactive prophylaxis compared to long-term administration of antibiotics for the prevention of recurrent urinary tract infections.

StroVac® (Strathmann, Hamburg, Germany) is a vaccine licensed in Germany for the prevention of recurrent urinary tract infections. A single dose of 0.5 ml contains a suspension of 10 strains of at least 10^9^ heat-inactivated bacteria: 6 different *E. coli* strains, and one strain of *Proteus mirabilis*, *Morganella morganii*, *Enterococcus faecalis*, and *Klebsiella pneumoniae* and aluminum phosphate as the adjuvant (4). In clinical studies, it effectively reduces recurrent urinary tract infections in women and men (1, 5–8). In general, women have a higher risk to develop recurrent urinary tract infections than men due to the anatomy of the female urinary tract and its proximity to the reproductive organs and the anus. The infection risk increases with age in women and men and especially in men age is a risk factor for the development of urinary tract infections (9).

The basic immunization consists of 3 intramuscular injections of StroVac® at an interval of 2 weeks. The vaccine is protective for approximately 12 months, thereafter a booster is necessary. Nestler et al., 2021 (1) compared the basic immunization plus one booster vaccination with StroVac® with the prophylactic use of nitrofurantoin for three months. Both StroVac® and nitrofurantoin protected patients against urinary tract infections within the first year of treatment. In the second year, patients clearly benefited from vaccination with StroVac®: 79.3% of the patients who had received the vaccine were protected against recurrent urinary tract infections, whereas only 59.2% of patients who had received nitrofurantoin did not develop a urinary tract infection (1). Moreover, two other studies demonstrated that within the first six months after vaccination with StroVac® the consumption of medications by patients with recurrent urinary tract infections was strongly reduced compared to an interval of 6 months before vaccination (6, 10).

The vaccine is considered to act via pathogen-directed protective antibodies, i.e., via the adaptive immune system (11). In heterologous transplant recipients, 3 doses of StroVac® did not induce donor-specific autoantibodies (7).

As bacterial components can directly stimulate receptors of the innate immune system and thereby activate cells (12) we analyzed the effect of StroVac® on phagocytes. Here, we demonstrate a direct effect of this bacterial suspension on macrophages in the absence of B- and T-lymphocytes, which is likely mediated by pathogen recognition receptors (PRR) of the innate immunity.

## Methods

### Bacterial growth curves

The *E. coli* strains 455 and 654 were grown in a medium containing 40 g/l yeast extract, 5 g/l glycerol, 3.75 g/l potassium dihydrogen phosphate, and 14 g/l di-sodium hydrogen phosphate for 72 h at 37°C under rotation (150 rpm) in the absence or presence of 0.03-30 mg/l StroVac®. At the indicated time points samples were taken, diluted in PBS and the number of bacteria was determined by quantitative plating on blood agar. Blood agar plates were incubated for 24 h at 37°C.

### Cell isolation

Primary bone-marrow derived macrophages (BMDM) were prepared from the femora of NMRI, C57BL/6, C3H/HeJ, or C3H/HeN mice and transferred into Dulbecco’s Minimal Essential Medium (DMEM) supplemented with 4.5 g/l D-glucose and L-glutamine. After centrifugation for 10 min at 4°C and 314 x G, the pellets were resuspended and cultured in medium containing 54% DMEM supplemented with 4.5 g/l D-Glucose, 30% cell culture supernatant of the cell line L929, 10% heat-inactivated FCS, 5% horse serum, 1% penicillin plus streptomycin (10.000 U/ml; 10.000 μg/ml) and 0.1% 2-mercaptoethanol for 6 days at 37°C in room air supplemented with 5% CO_2_. On the seventh day after preparation, macrophages were differentiated into pro- (M1, addition of 0.1 mg/l IFNγ for 20 h) or anti-inflammatory (M2, addition of 0.001 mg/l IL-4, 0.001 mg/l IL-10 and 0.001 mg/l IL-13 for 20 h) phenotypes or left undifferentiated (M0) (13).

The murine macrophage and monocyte cell line J774A.1(ATCC 2022) stems from a reticulum cell sarcoma, is active in phagocytosis, and was cultured in DMEM supplemented with 1 g/l D-glucose, 10% heat-inactivated FCS and 100 U/ml penicillin plus 100 μg/ml streptomycin (Gibco, Karlsruhe, Germany).

### Stimulants

The 10 bacterial strains constituting the vaccine StroVac® (≥10^9^ bacteria/0.5 ml) (4) were heat-inactivated at 65 ± 3°C for 90 – 100 min (*Escherichia coli, Proteus mirabilis, Morganella morganii*, and *Klebsiella pneumoniae*) or 76 ± 2 C° (*Enterococcus faecalis*) and then incubated for 4 weeks in phosphate-buffered saline (PBS) containing 0.35% phenol as part of the inactivation and manufacturing process. Twenty-one mg of StroVac® dry matter was suspended in 0.5 ml of basic suspension containing the adjuvant. The concentration of the adjuvant aluminum phosphate in this suspension was 1.0 mg per vaccine dose. The concentration of endotoxin was 1470 IU/ml (manufacturer`s information). The endotoxin content in the whole cell vaccine originates from the Gram-negative bacteria incorporated in the vaccine. StroVac® was used at final concentrations from 0.3 to 300 mg/l. There is no close correlation between the number of bacteria and the extract in mg described. A single dose of the vaccine (21 mg dry matter suspended in 0.5 ml) contains at least 10^9^ inactivated bacteria.

Assuming a volume of distribution of 1 l/kg, a concentration of 0.3 mg/l can be reached *in vivo* in humans after vaccination. As the vaccine probably does not distribute equally in the body, we tested different concentrations of StroVac® *in vitro*. Concentrations higher than 30 mg/l were not regularly tested as they induced cytotoxic effects *in vitro*.

LPS from *E. coli* O111:B4 purchased from Merck (Darmstadt, D) served as positive control at a concentration of 0.01 and 0.1 mg/l (14).

The Toll-like receptor agonist CpG ODN 1668 (5′ TCC ATG ACG TTC CTG ATG CT, molecular mass 6382.6 g/mol, TIB Molbiol, Berlin, Germany) served as a positive control in phagocytosis experiments at a concentration of 1.0 mg/l (14).

### Cytokine release and phagocytosis

Fifty-thousand BMDM (M0, M1, or M2 macrophages) or J774A.1 cells per well in a 96-well plate were stimulated with StroVac® or the basic suspension of the vaccine containing the adjuvant (only J774A.1) at concentrations from 0.3 to 30 mg/l and in some cases 0.3 to 300 mg/l for 24 h in RPMI supplemented with 10% heat-inactivated FCS (BMDM) or DMEM containing 1.0 g/l D-Glucose supplemented with 10% heat-inactivated FCS (15).

In order to quantify phagocytosis, 5x10^6^ colony-forming units (CFU) *E. coli* DH5-α or *Streptococcus (S.) pneumoniae* R6, two unencapsulated apathogenic strains kindly provided by Prof. Sven Hammerschmidt, Dept. of Molecular Genetics and Biology of Infection, University Greifswald, Germany (16), were co-incubated with BMDM for 45 min in RPMI medium 1640 supplemented with 10% heat-inactivated fetal calf serum (FCS). Bacteria and J774A.1 cells were co-incubated in DMEM with 1.0 g/l D-glucose, and pyruvate supplemented with 10% heat-inactivated FCS for 45 min. Thereafter, macrophages and bacteria were centrifuged for 10 min at 314 x G at room temperature, and the supernatants were used to determine the concentrations of the cytokines TNF-α, IL-6, IL-12/IL-23 p40, and IL-1β by enzyme-linked immunosorbent assay (ELISA Deluxe Kits, BioLegend, Amsterdam, Netherlands) following the manufacturer`s instructions. The detection limit of the assays was 3.9 ng/l (TNF-α, IL-6, IL-12/IL-23 p40 and IL-1β). Cell viability was determined by the concentration of lactate dehydrogenase (LDH) in the supernatants using 150000 J774A.1 cells and following the manufacturer`s instructions (CytoTox-ONE, Promega, Walldorf, Germany).

After washing the phagocytes with PBS, the bacteria and eukaryotic cells were co-incubated in DMEM or RPMI medium containing 10% FCS and 100 mg/l gentamicin to kill extracellular bacteria for 45 min. After another washing step with PBS, eukaryotic cells were lysed with 100 µl distilled water, and the numbers of intracellular (phagocytosed) bacteria were determined by quantitative plating on blood agar after incubation at 37°C for 24 h. The number of intracellular bacteria was determined by counting the colony-forming units.

### Statistics

To compare phagocytosis experiments performed on different days, all data were normalized to the respective control groups. Since in most cases, data were not normally distributed, the medians and 25th/75th percentiles were shown. The median number of phagocytosed bacteria in the absence of StroVac® (or the basic suspension), LPS (control) and CpG (control) was set at 100% and the phagocytosed bacteria in the presence of StroVac®, LPS and CpG were expressed as a percent of the median of the respective control group. Groups were compared by Kruskal-Wallis test followed by Dunn’s multiple comparisons test to correct for repeated testing, and an α error ≤ 0.05 was considered statistically significant. The LDH assay was analyzed using one-way ANOVA followed by Dunnett’s multiple comparisons test as the post-test.

For the assessment of the maximum effect (maximum phagocytosis expressed in % of the phagocytosis of unstimulated cells, E_max_) and of the concentration causing 50% of the maximum effect (EC_50_), a sigmoid dose-response curve was constructed using the medians of the phagocytosis (in percent) at the respective concentrations.

All calculations were performed with Graph Pad Prism, Version 6.01 (GraphPad Software, San Diego, CA, USA).

## Results

**Figure 1:**
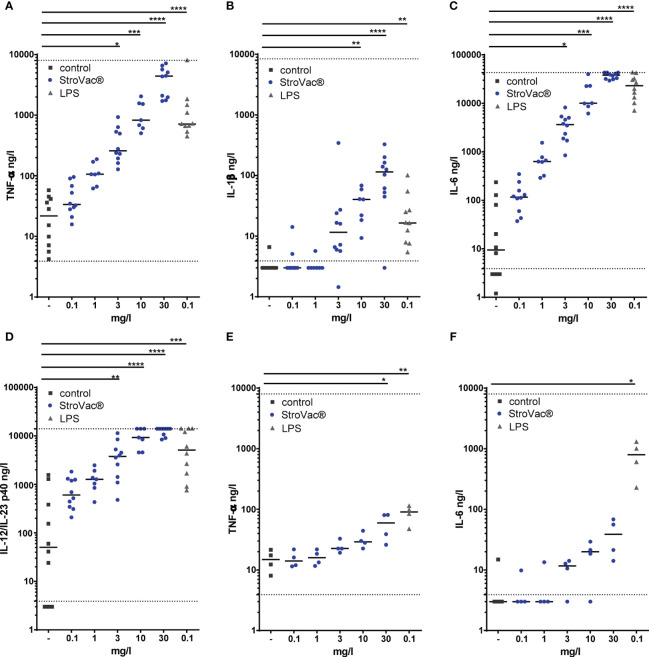
Release of pro-inflammatory cytokines by primary murine bone-marrow derived macrophages (BMDM isolated from C57BL/6 mice) (**A–D**; TNF-α: **A**, IL-1ß: **B**, IL-6: **C**, IL-12/IL-23 p40: **D**) and the J774A.1 macrophage cell line (**E**, **F**; TNF-α: **E**, IL-6: **F**). Primary macrophages and J774A.1 cells were stimulated by different concentrations of the inactivated bacterial vaccine StroVac® (0.3 mg/l – 30 mg/l) or 0.1 mg/l LPS as positive control for 24 h. Fifty-thousand cells were used per well. The horizontal bars represent the medians (4 – 10 measurements from 4 – 6 independent experiments (*p<0.05, **p<0.01, ***p<0.001, ****p<0.0001; Kruskal-Wallis test followed by Dunn’s multiple comparisons test). The dotted lines represent the limits of detection.

In the absence of eukaryotic cells, the presence of StroVac® did not influence bacterial growth (**Supplemental Figure 1**). Upon stimulation by StroVac®, primary murine macrophages released the cytokines TNF-α, IL-6, IL-12/23 p40, and IL-1β in a dose-dependent manner (**Figures 1A–D**). The J774A.1 macrophage cell line in response to stimulation by StroVac® released TNF-α but did not release significant amounts of IL-6 (**Figures 1E, F**). No release of IL-12/23 p40 and IL-1β was detectable after stimulation with StroVac® or LPS (data not shown). Stimulation of J774A.1 macrophage with the basic suspension (containing the adjuvant and all other components except the bacteria) induced a small dose-dependent release of TNF-α (**Supplemental Figure 2**).

**Figure 2:**
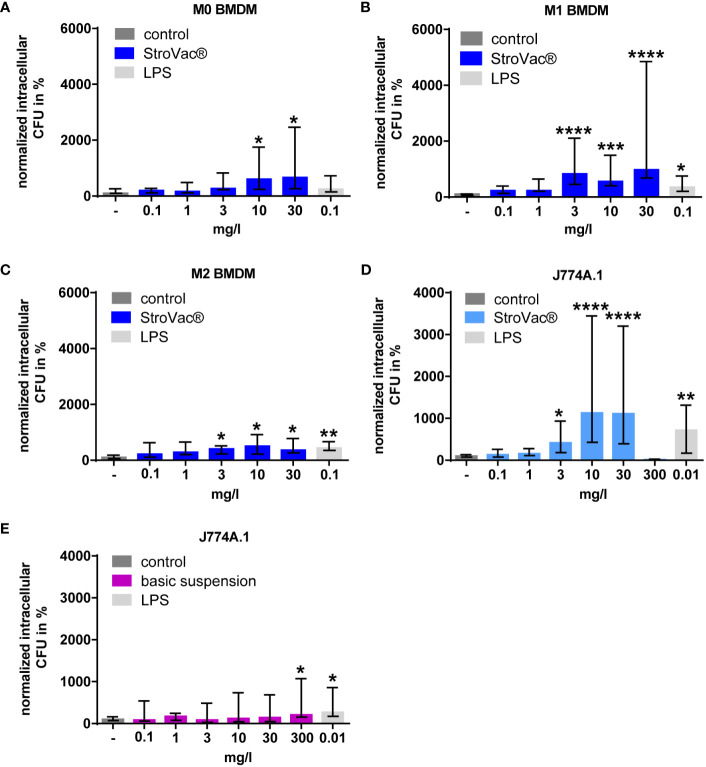
Phagocytosis of *E. coli* by primary murine bone-marrow derived macrophages (BMDM isolated from C57BL/6 mice, **A–C**) and the J774A.1 macrophage cell line **(D, E)**. Primary macrophages [undifferentiated = M0 **(A)**, pro-inflammatory = M1 **(B)**, anti-inflammatory = M2 **(C)**] and J774A.1 cells **(D)** were stimulated by different concentrations of the inactivated bacterial vaccine StroVac® **(A–D)** or the basic suspension **(E)** or LPS as positive control for 24h. Data are expressed as medians (25th/75th percentiles). Fifty-thousand cells per well were used. Median phagocytosis of unstimulated cells in each individual experiment was defined as 100% (3 – 12 measurements from 3 – 4 independent experiments) (*p<0.05, **p<0.01, ***p<0.001, ****p<0.0001; Kruskal-Wallis test followed by Dunn’s multiple comparisons test).

In bone marrow-derived macrophages, StroVac® stimulated phagocytosis of *E. coli* and *S. pneumoniae* in a dose-dependent manner (**Figure 2** and **Supplemental Figure 3**). This increase of phagocytosis was observed in primary M0, M1, and M2 macrophages, i.e., at different activation statuses of the macrophages (**Figures 2A–C**), and in the murine macrophage cell line J774A.1 (**Figure 2D** and **Supplemental Figure 3**). The EC_50_ was 5.12 mg/l in primary M0, 1.21 mg/l in M1, 0.67 mg/l in M2 cells and 3.56 mg/l in J774A.1 cells, respectively. The E_max_ was 673% in primary M0, 789% in M1, 436% in M2 cells and 1122% in J774A.1 cells.

**Figure 3:**
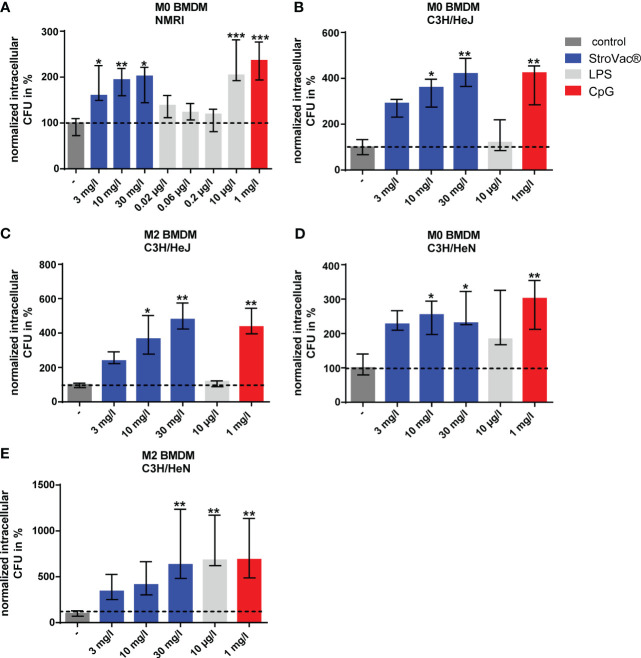
Phagocytosis of *Escherichia coli* by primary murine bone-marrow derived macrophages (BMDM). BMDM [undifferentiated = M0 **(A, B, D)**, anti-inflammatory = M2 **(C, E)**] isolated from NMRI **(A)**, C3H/HeJ **(B, C)** or C3H/HeN **(D, E)** mice were stimulated by different concentrations of the inactivated bacterial vaccine StroVac®, the LPS content of the respective StroVac® concentrations (0.02 – 0.2 µg/l) or 10 µg/l LPS as positive control for 24 h. CpG was used as a positive control for the BMDM of the LPS-tolerant mouse strain C3H/HeJ. Data are expressed as medians (25th/75th percentiles). 4 – 6 measurements from 2 independent experiments (*p<0.05, **p<0.01, ***p<0.001, Kruskal-Wallis test followed by Dunn’s multiple comparisons test).

The stimulation of bacterial phagocytosis was independent of the LPS content of StroVac®. LPS concentrations equivalent to the LPS content of 3, 10, and 30 mg/l StroVac® did not stimulate phagocytosis of BMDM isolated from wild-type mice. Moreover, BMDM of the LPS-tolerant mouse strain C3H/HeJ showed increased phagocytosis of *E. coli* after stimulation with StroVac®, but not after stimulation with LPS. In line with these results, BMDM isolated from the LPS-sensitive mouse strain C3H/HeN phagocytosed significantly more bacteria after stimulation with LPS compared to the control. The Toll-like receptor agonist CpG served as a positive control for stimulation of phagocytosis. The results showed that the bacterial components of StroVac® stimulate innate immune cells independent of the LPS content of StroVac® (**Figure 3**).

Up to a concentration of 30 mg/l, the adjuvant did not stimulate the phagocytosis of bacteria by the J774A.1 macrophage cell line. At 300 mg/l, the adjuvant caused a small, but significant increase of the phagocytic activity of J774A.1 macrophages (n = 15, increase of 207%, p=0.0498, Kruskal-Wallis test followed by Dunn’s multiple comparisons test) (**Figure 2E**).

**Figure 4:**
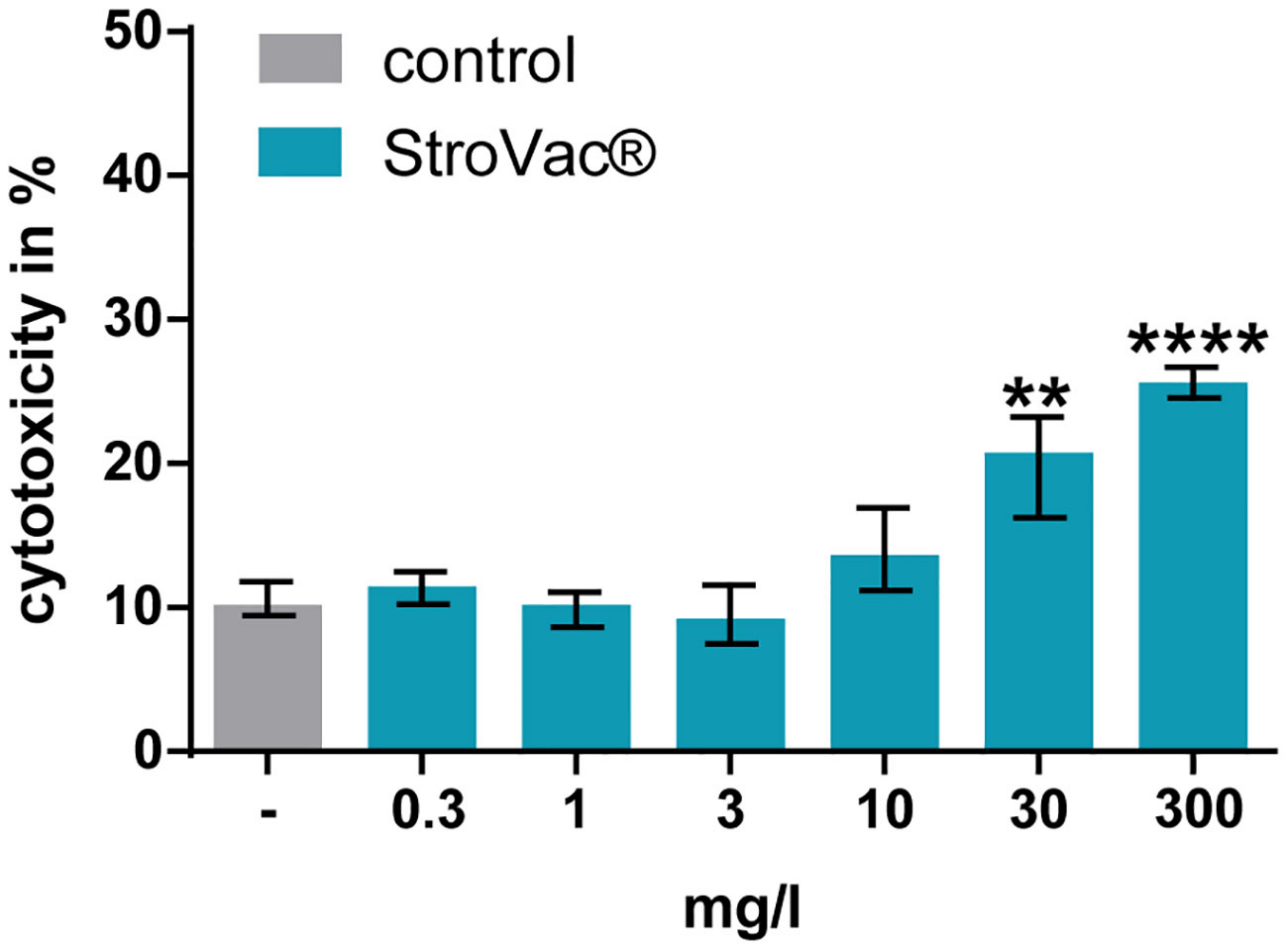
Cytotoxic effect of StroVac® on J774A.1 macrophages. 0.3 – 300 mg/l StroVac® were incubated with 150000 cells of the cell line J774A.1 for 24 hours. LDH release was measured and normalized to completely lysed cells. Complete lysis was defined as 100% (3 measurements) (**p<0.01, ****p<0.0001; one-way ANOVA followed by Dunnett’s multiple comparisons test). Please note moderate (at 30 mg/l) and strong (at 300 mg/l) toxicity of StroVac® to eukaryotic cells.

LDH concentrations measured in the cell culture supernatants suggested that StroVac® was not toxic to eukaryotic cells up to a concentration of 10 mg/l. Only at the concentration of 30 and 300 mg/l of StroVac®, an increase of the LDH concentrations in cell culture supernatants was detected, suggesting toxicity of the vaccine to eukaryotic cells at very high concentrations presumably as a consequence of excessive stimulation of phagocytes (**Figure 4**).

The absence of phagocytosis at 300 mg/l in J774A.1 macrophages is caused by direct toxicity of StroVac® to eukaryotic cells (**Figure 4**). In such a high, non-physiological concentration, the vaccine had a cytotoxic effect. Concentrations occurring in human plasma and other body fluids after vaccination are several orders of magnitude lower than the concentrations causing *in-vitro* toxicity.

## Discussion

The inactivated bacterial suspension StroVac® used as a vaccine for the prevention of urinary tract infections caused a dose-dependent increased release of pro-inflammatory cytokines.

The cytokines TNF-α, IL-6, IL-12/IL-23 p40, and IL-1β were measured in this experimental set-up as they are highly important for the defense against a bacterial infection: the release of TNF-α increases phagocytic capacities of macrophages (17). IL-6 mediates innate and adaptive immunity, as it is produced by innate immune cells, promotes the differentiation of B-lymphocytes to immunoglobulin-producing cells, and regulates the differentiation of naïve CD4+ T-Zellen (18, 19). Moreover, it contributes to the host’s resistance to bacterial infections. IL-1ß induces fever, the release of proinflammatory molecules, and stimulates the innate immune system (20–22). IL-12 is highly important for the infection resistance of the host and induces stimulation of TH1 cells (23, 24). IL23 regulates the TH17 cell response (25).

The cytokine release of cell line J774A.1 compared to that of primary macrophages was comparably low. TNF-α and IL-6 were detectable after stimulation with StroVac®, but the measured values for IL-12/IL-23 p40 und IL-1β were below the detection limit of 3.9 pg/ml. It is well known that cell lines differ from primary cells regarding their phenotype and gene expression. Due to immortalization and repeated subculturing of cells, the phenotype might be altered (26).

Analysis of the concentration-response curves of the phagocytic activity demonstrated that E_max_ was high in M0 and M1 primary macrophages isolated from C57BL/6 mice and J774A.1 cells and comparably low in M2 primary macrophages. Conversely, the EC_50_ was lowest in M2 cells. This compares well with the behavior of macrophages prepared from the blood of eight healthy blood donors *ex vivo*: M1 cells exhibited the highest level of phagocytic activity of FITC-labeled *E. coli*, but phagocytic activity also was observed in M0 and M2 macrophages (27).

Incubation of primary macrophages with StroVac® induced a concentration-dependent release of the pro-inflammatory cytokines TNF-α, IL-6, IL-12/IL-23 p40, and IL-1β. In the absence of lymphocytes, this stimulatory effect is most probably caused by the activation of the innate immune system. In this respect, lipopolysaccharides as Toll-like receptor (TLR)-4 ligands appear not to play a major role. Almost all lipopolysaccharides were destroyed by heat during the preparation of the vaccine, and the maximum concentrations of LPS present in the vaccine according to information of the manufacturer did not stimulate eukaryotic cells in our experimental setting. Moreover, StroVac® stimulated phagocytosis in C3H/HeJ BMDM, which are resistant to the stimulatory effect of LPS. Therefore, components other than LPS of this inactivated bacterial suspension strongly stimulated the innate immune system. Immune cells can detect conserved molecular signatures [pathogen-associated molecular patterns (PAMPs) or damage-associated molecular patterns (DAMPS)] through pattern recognition receptors (PRR) (28). Five different classes of PRR have been described: Toll-like receptors (TLR) and C-type lectin receptors sense PAMPS and DAMPS situated extracellularly or within vacuolar compartments. NOD-like receptors, AIM2-like receptors, and retinoic acid-inducible gene (RIG)-I-like receptors recognize PAMPS and DAMPS located within the cytoplasm. Activation of PRR can result in a pro-inflammatory response by nuclear factor kB (NFkB) or interferon-regulatory factors (IRFs) pathways or by activation of inflammasomes, macromolecular complexes which assemble in the cytosol triggering a proinflammatory response (28). In addition, adjuvants such as aluminum salts, saponins, and emulsions which have the function to trigger the innate immune system to intensify signal transition to the adaptive immune system can activate inflammasomes (29). Therefore, it is very likely that different components of StroVac® other than LPS activate innate immune cells. Studies by Elson et al., 2007 (12) proved that viable as well as heat-inactivated Gram-positive and Gram-negative bacteria are able to activate cells via different innate immune receptors.

Locally synthesized secretory IgA was shown to be low in women with recurrent infection of the urinary tract and these low antibody levels may predispose to recurrent urinary tract infections (30). The reported effect of this vaccine is the reduction of the frequency of recurrent urinary tract infections via increasing urinary IgA production (11). Our data, demonstrating stimulation of macrophages in the absence of lymphocytes, suggest an additional activation of the antimicrobial capabilities of phagocytes by the innate immune system as an integral part of the action of this vaccine. The long-term memory of the innate immune system has been studied in the last few years. It has been shown that the innate immune system exhibits memory-like capacities termed “trained innate immunity” (31). These long-term effects of trained innate immunity are mediated by epigenetic and metabolic changes and may lead to a long-term enhanced immune response against the same or different pathogens (32, 33). In recent years, a multitude of convincing data have been published on the effects of vaccines and bacterial components, e.g. the BCG vaccine which does not only protect against an infection with tuberculosis but also leads to a robust non-specific protection against other infections mediated by innate immune cells such as monocytes, macrophages, and natural killer cells (34). Resistance against a heterologous infection induced by live vaccines was shown to last for up to 5 years. The immunological phenotype of trained innate immunity is traceable depending on the study for 3 months up to one year (summarized by (33)).

One limitation of our study is that we did not analyze the phenotype of the innate immune cells after stimulation with StroVac® with respect to TLR expression or expression of activation markers of innate immune cells on RNA or protein level. We hypothesize that the concept of trained innate immunity might be involved in the protective effect of StroVac® against infections, but clearly, *in-vivo* studies need to be performed to prove this hypothesis. We plan to perform *in-vivo* experiments in which mice lacking B- and T-cells will be immunized with StroVac® and then analyzed for the duration of their immune response and their protection against infections. Moreover, macrophages will be isolated from immunized wild-type mice at different time points and analyzed with respect to their antibacterial activity and cytokine production. Investigating the impact of StroVac® on the protection against heterologous infections is a further important goal of future studies. Already published data suggest long-term protection by trained innate immunity: two intra-peritoneal injections of 1 mg of the Toll-like receptor-2 agonist zymosan at an interval of 4 days protected mice from *Listeria monocytogenes* infections for 9 weeks (35). Induction of trained innate immunity by zymosan or *Candida albicans* protected the descendants of trained mice against systemic heterologous *E. coli* and *Listeria monocytogenes* infections (36).

In conclusion, StroVac® does not only act via the adaptive but also via the innate immune system. The exact mechanisms and the duration of the stimulatory effect on the innate immune system remain to be determined. In clinical practice, this vaccine helps to reduce the consumption of antibiotics in urinary tract infections and may be effective in other bacterial infections caused by the pathogens incorporated in this vaccine. It therefore may be an aid to decrease the evolutionary pressure on bacteria by the excessive use of antibiotics.

## Data availability statement

The raw data supporting the conclusions of this article will be made available by the authors, without undue reservation.

## Ethics statement

The animal study was reviewed and approved by Landesamt für Verbraucherschutz und Lebensmittelsicherheit Niedersachsen.

## Author contributions

AE conducted experiments, analyzed data and drafted the manuscript. MB and BM conducted experiments. RN supervised and designed the study and drafted the manuscript. JS contributed to study design, performed experiments, analyzed data and drafted the manuscript. All authors read and approved the final version of the manuscript.
