# Supplementary material for: A suspension of inactivated bacteria used for vaccination against recurrent urinary tract infections increases the phagocytic activity of murine macrophages
Source: Front Immunol. 2023 Aug 16;14:1180785. doi: 10.3389/fimmu.2023.1180785 (PMC10467261; doi:10.3389/fimmu.2023.1180785)
Supplement: Supplementary file 1 [file DataSheet_1.docx]

Supplementary Material

A suspension of inactivated bacteria used for vaccination against recurrent urinary tract infections increases the phagocytic activity of murine macrophages

Anja Eggers^1,2^, Melissa Ballüer^1,2^, Belal A. Mohamed^3,4^, Roland Nau^1,2^, Jana Seele^1,2^

^1^Department of Geriatrics, Evangelisches Krankenhaus Göttingen-Weende, Göttingen, Germany; ^2^Department of Neuropathology, University Medical Center Göttingen, Göttingen, Germany; ^3^ Department of Cardiology and Pneumology, University Medical Center Göttingen, Göttingen, Germany; ^4^DZHK (German Centre for Cardiovascular Research), Göttingen, Germany.


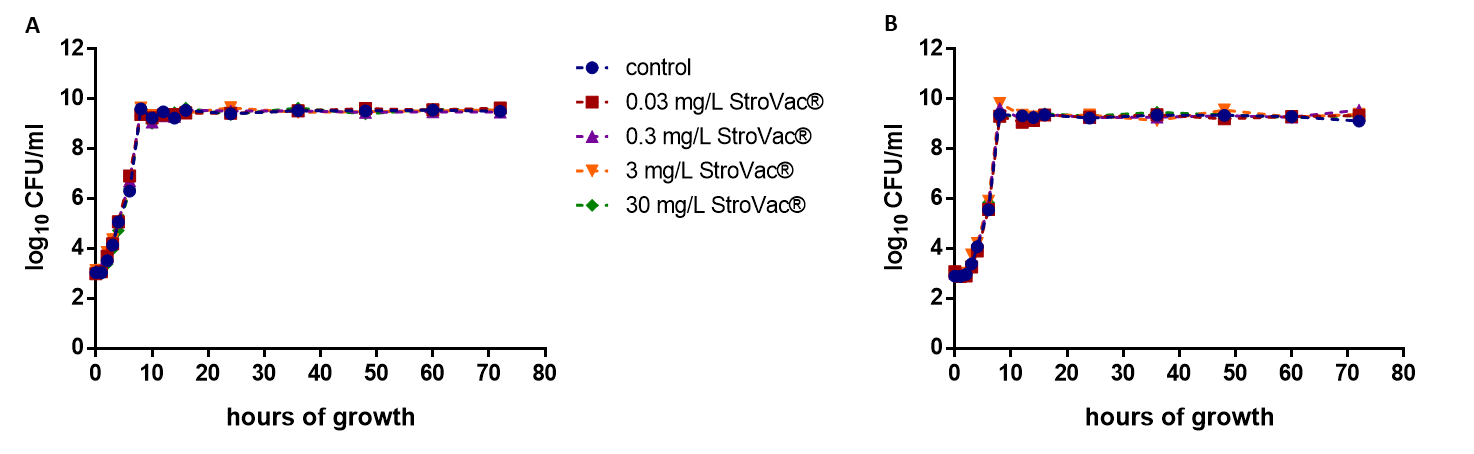


Supplemental Figure 1: Growth curves of *E. coli* 455 (A) and 654 in the presence of 0.03-30 mg/l StroVac®. Bacteria were grown for 72h at 37°C with rotation (150 rpm). Three independent experiments are shown. At all concentrations studied, StroVac® did not influence bacterial growth.

Supplemental Figure 2: Release of the pro-inflammatory cytokine TNF-α by J774A.1 macrophages. J774A.1 cells were stimulated by different concentrations of the basic suspension (0.3 mg/l – 30 mg/l) or 0.1 mg/l LPS as positive control for 24 h. Fifty-thousand cells were seeded per well (4 – 10 measurements from 4 – 6 independent experiments; ***p<0.001, ****p<0.0001; Kruskal-Wallis test followed by Dunn’s multiple comparisons test). The dotted lines represent the limit of detection.

Supplemental Figure 3: Phagocytosis of *S. pneumoniae* R6 by the macrophage cell line J774A.1. Cells were stimulated with different concentrations of the inactivated bacterial vaccine StroVac® (0.3 mg/l – 30 mg/l) or 0.01 mg/l LPS as positive control for 24h. Data are expressed as medians (25th/ 75th percentiles). Fifty-thousand cells per well were used. Median phagocytosis of unstimulated cells in each individual experiment was defined as 100% (7 - 8 measurements from 2 independent experiments) (*p<0.05, ****p<0.0001; Kruskal-Wallis test followed by Dunn’s multiple comparisons test).
